# Expression and clinical implications of basic leucine zipper ATF-like transcription factor 2 in breast cancer

**DOI:** 10.1186/s12885-021-08785-6

**Published:** 2021-09-26

**Authors:** Yingying Lin, Xusheng Zhou, Wei Peng, Jing Wu, Xiufeng Wu, Yan Chen, Zhaolei Cui

**Affiliations:** 1 Laboratory of Biochemistry and Molecular Biology Research, Department of Clinical Laboratory, Fujian Medical University Cancer Hospital, No. 420 Fuma Road, Jin’an District, Fuzhou, 350014 Fujian Province China; 2 Department of Breast Surgical Oncology, Fujian Medical University Cancer Hospital, Fuzhou, Fujian China

**Keywords:** BATF2, Breast cancer, Bioinformatics, Serum, Exosomes, Biomarker

## Abstract

**Background:**

Basic leucine zipper ATF-like transcription factor 2 (BATF2) has been reported to participate in the occurrence and development of some malignancies. Herein, we aimed to explore the expression pattern and clinical implications of BATF2 in breast cancer (BC).

**Methods:**

We assessed the differences in *BATF2* mRNA expression between cancerous and noncancerous tissues in BC using GEPIA and UALCAN data and in BATF2 protein expression pattern using Human Protein Atlas (HPA) data. *BATF2* co-expression networks were analyzed in Coexpedia. The association between the differentially expressed *BATF2* mRNA and BC prognosis was assessed using UALCAN, OSbrca, and GEPIA databases. In external validations, BATF2 protein expression in BC tissues was quantitated using a tissue microarray and immunohistochemistry (IHC) analysis, and *BATF2* mRNA expression in serum and serum-derived exosomes of BC patients using real-time quantitative reverse transcription polymerase chain reaction (qRT-PCR).

**Results:**

No difference in the *BATF2* mRNA expression level was found between cancerous and noncancerous tissues in BC based on databases. There were low-to-moderate levels of increases in BATF2 protein expressions in BC cases from the HPA cohort. *BATF2* mRNA expression was negatively correlated with androgen receptor (*AR*) and positively correlated with *BRCA2 DNA repair associated (BRCA2)*, *marker of proliferation Ki-67* (*Mki67)*, and *tumor protein p53* (*TP53)* expressions. Generally, *BATF2* mRNA exhibited a non-significant association with BC prognosis; yet the subgroup analyses showed that triple-negative breast cancer (TNBC) patients with high *BATF2* mRNA expressions had a longer overall survival (OS). Our IHC analysis revealed a positive rate of BATF2 protein expression of 46.90%, mainly located in the nucleus of cancer cells in BC, and the OS of BC patients with high BATF2 protein expressions was prolonged. The positive rates of *BATF2* mRNA expressions in the serum and exosomes were 45.00 and 41.67%, respectively. Besides, the AUCs of serum and exosomal *BATF2* mRNA for BC diagnosis were 0.8929 and 0.8869, respectively.

**Conclusions:**

BC patients exhibit low-to-moderate expressions in *BATF2* mRNA expression levels in cancerous tissues. The high BATF2 protein expression can be a potential indicator of a better BC prognosis. Serum and exosomal *BATF2* mRNA levels also serve as promising noninvasive biomarkers for BC diagnosis.

**Supplementary Information:**

The online version contains supplementary material available at 10.1186/s12885-021-08785-6.

## Background

BC is one of the most prevalent malignancies in women [1]. The latest cancer statistics showed that BC topped the list of cancer morbidity in Chinese women, with a mortality rate ranking fourth [2]. Most studies agree that intricate multi-gene networks are involved in the occurrence, development, and metastasis of BC in synergy, accompanied by mutations and/or abnormal genetic activities, such as proto-oncogene activation, apoptosis inhibition of tumor suppressor genes, abnormalities in gene expressions [3–5]. For personalized diagnosis, treatment, and prognosis evaluation of BC, arduous tasks are ahead of us to unveil the underlying genetic mechanisms behind the onset and evolution of BC.

*BATF-2*, also known as suppressor of activator protein-1 regulated by interferon (*SARI*), is a recently discovered tumor suppressor gene using subtractive hybridization in 2008 [6]. It is a type I interferon (IFN) inducible protein with a leucine zipper and an activator protein (AP)-1 transcription factor family member and has specific structural characteristics to activate transcription factors [6]. Pieces of evidence show that the BATF-2 gene, though expressed in a variety of normal tissue cells (e.g., melanocytes, astrocytes, pancreatic mesothelial cells, and prostate epithelial cells), can selectively inhibit the growth of tumor cells [6, 7]. Strikingly, recent studies found its roles in the occurrence and development of various malignancies. For example, *BATF-2* mRNA expression is down-regulated in chronic myeloid leukemia (CML) patients compared to healthy individuals, and BCR-ABL chimeric protein participates in the inhibition of *BATF-2* gene expression [8]. Abnormalities in BATF-2 and cellular communication network factor 1 (CCN1) expressions and their correlation are closely associated with malignant behaviors of colorectal cancer cells, affecting the prognosis of patients [9, 10]. Moreover, in other tumors such as hepatocellular carcinoma (HCC) [11], non-small-cell lung cancer (NSCLC) [12], esophageal squamous cell carcinoma (ESCC) [13] and gastric cancer (GC) [14], the down-regulation of BATF-2 expression is associated with a poor prognosis. Wang et al. found that BATF-2 regulated the epithelial-mesenchymal transition (EMT) and lung adenocarcinoma (LUAD) metastasis [15]. However, little is known about the roles of BATF-2 in BC. Studies on the expression patterns and clinical implications of BATF-2 in BC are needed.

In contrast to preclinical and clinical studies that are time-consuming, bioinformatics has provided convenience or high efficiency for studies of genetic activity in cancers since the Human Genome Project paved the way [16]. As gene and protein reaction networks consist of voluminous interactions, bioinformatics is a useful tool for the studies of genomics, proteomics, and other fields [17]. In this work, we sought to explore the expression patterns and diagnostic and prognostic implications of *BATF2* mRNA and protein expressions in BC using bioinformatics, which were subsequently verified in the serum, serum-derived exosomes, and cancer tissues of BC patients using qRT-PCR and IHC.

## Methods

### Study design

We first assessed the difference in *BATF2* mRNA expression between cancerous and noncancerous tissue samples from GEPIA [18] and UALCAN [19] databases, and that in BATF2 protein expression between the two cohorts of samples in HPA. The gene co-expression network of *BATF2* was plotted using Coexpedia [20], and the correlations between *BATF2* mRNA and other co-expressed markers (e.g., HER2 and BRCA2) in BC were evaluated using GEPIA. The prognostic value of* BATF2 *mRNA expression in BC prognosis was assessed using OSbrca [21], UALCAN, and Kaplan-Meier Plotter [22]. Based on the bioassay data, we further set up tissue microarray and IHC analysis to verify the BATF2 protein expression pattern in the serum and serum-derived exosomes of BC patients and its correlation with clinicopathological features and prognosis. Serum and exosomal *BATF2* mRNA expressions were determined using qRT-PCR analysis.

### Clinical data

We performed protein expression analysis of BATF2 using clinical data of a high-throughput tissue microarray (HBreD145Su02, SHANGHAI OUTDO BIOTECH) consisting of 145 cancer tissues and 90 adjacent noncancerous tissues (SHANGHAI OUTDO BIOTECH) from 145 female patients who were pathologically diagnosed and underwent radical mastectomy from August 2004 to December 2008. Their medical records were complete and available. These patients were aged from 33 to 88 years, with an average age of 58 years. As for BC subtypes, one case was diagnosed with intraductal carcinoma, 2 with mucinous carcinoma, 4 with invasive lobular carcinoma, 119 with invasive ductal carcinoma, 9 with invasive micropapillary carcinoma, 3 with invasive lobular carcinoma, 2 with medullary carcinoma, and 5 with mucinous carcinoma. These patients did not receive radiotherapy, chemotherapy, or other tumor-related treatments before the operation. Also, 60 serum samples of BC patients hospitalized in Fujian Cancer Hospital from December 2018 to May 2019 were collected, and 56 serum samples of normal healthy people were collected as controls. All included BC individuals were invasive ductal carcinoma and non-TNBC (triple-negative breast cancer) cases, with an average age of 56 years. All serum specimens were collected with the approval of the Ethics Committee of Fujian Medical University Cancer Hospital (ethical approval certificate: No. SQ2018–015-01).

### Public data and bioinformatics tools

GEPIA is a tumor analysis webserver for assessing mRNA expression data, other gene expression profiles, differentially expressed genes and survival of patients with various tumors and their subtypes using TCGA and GTEx data [18]. UALCAN database offers gene expression analysis and survival analysis based on clinical data from TCGA [19]. In this study, both GEPIA and UALCAN databases were utilized to appraise the difference in *BATF2* mRNA expression between cancerous and noncancerous tissues in BC. HPA database provides information on the tissue and cell distribution of 26,000 human proteins and the expression of each protein in 64 cell lines across 48 human normal tissues and 20 tumor tissues. We extracted data about the expression of BATF2 protein and clinicopathological features of BC tissues from HPA. The gene co-expression network of *BATF2* together with other target genes was depicted using Coexpedia [20] to predict relevant diseases or pathways enriched in target genes. OSbrca a professional prognostic tool with comprehensive data sources and large sample size (2277 malignancies and 31,310 patients from 209 data sets derived from TCGA, GEO, CGGA, and other databases). Kaplan-Meier Plotter to evaluate mRNA, miRNA, and protein expressions on survival supports the multi-gene query and pan-cancer analysis of 21 tumors. We analyzed the correlations between *BATF2* mRNA expression and BC prognosis using OSbrca [21], Kaplan-Meier Plotter [22], GEPIA, and UALCAN databases and appraised its prognostic effect based on the prognosis-survival curve.

### IHC analysis

The BATF2 protein in paraffin-embedded BC tissues was identified by the rabbit anti-human BATF2 polyclonal antibody (Abcam, Catalog No.ab204510; 1:50) using the EliVisionTM Plus two-step detection system according to its protocol. PBS was used instead of the primary antibody as a negative control. The experimental results were judged to be completed under the guidance of clinically experienced pathologists. The scoring criteria were based on our previously published articles [23, 24].

### Isolation and identification of exosomes

Exosomes were extracted from serum samples using an exoRNeasy Serum/Plasma Midi Kit (QIAGEN, Catalog No.77044), as described in the manufacturer’s protocol (www.qiagen.com/hb-1179). The extracted exosome vesicles were determined by transmission electron microscopy (TEM). As for the quantification of BATF2 protein expression, samples were lysed by RIPA lysis to extract the total protein. The equivalent amount of sample proteins were loaded onto each well for western blot analysis, and samples were incubated with mouse anti-human GAPDH monoclonal antibody (1:1000) and rabbit anti-human monoclonal CD63 (Abcam, Catalog No.ab217345) and CD9 (Abcam, Catalog No.ab92726) primary antibodies (1:100), visualized and photographed. According to The International Society for the extraction of extracellular vesicles [25], the rabbit anti-human Cytochrome C monoclonal antibody (Beyotime, Catalog No.AF2047; 1:100) was utilized as a negative control. The specific procedures had been described in our previous study series [23, 24].

### Real-time quantitative reverse transcription polymerase chain reaction (qRT-PCR)

The total RNA from the serum and serum-derived exosomes was extracted using a miRNeasy Kit and exoRNeasy Serum/Plasma Midi Kit according to the manufacturer’s protocol. The extracted total RNA was reverse transcribed into cDNA using the Transcriptor First-strand cDNA synthesis kit (Roche). The quantitative real-time PCR analysis was performed in the ABI7500 fluorescence quantitative PCR detector using SYBR Green Master (ROX) Mix. The primer sequences of the target products incorporated *BATF2*-F: 5′-GCCTAAGCCATGCACCTCTGT-3′, *BATF2*-R: ‘-TCTTCAGCTGCCTTTGTTGCTC -3’, *GAPDH*-F: 5′-GGAGCGAGATCCCTCCAAAAT-3′, and *GAPDH*-R: 5′-GGCTGTTGTCATACTTCTCATGG-3′ [23, 24]. The amplification was carried out as follows: initial denaturation at 95 °C for 10 min, 40 cycles of amplification at 95 °C for 15 s, and extension at 60 °C for 1 min. The expression level of BATF2 mRNA relative to the internal reference gene was evaluated using the 2^-ΔΔCT^ method.

### Statistical analysis

SPSS 16.0 software was employed for all statistical analyses. The relative experimental values were expressed as mean ± standard deviation (SD). After the homogeneity test for variance, the comparisons of differences between groups were accomplished using the Student’s t-test, and two-side *P* < 0.05 was assumed as statistical significance.

## Results

### Patterns of *BATF2* mRNA and protein expressions in BC

**Fig. 1:**
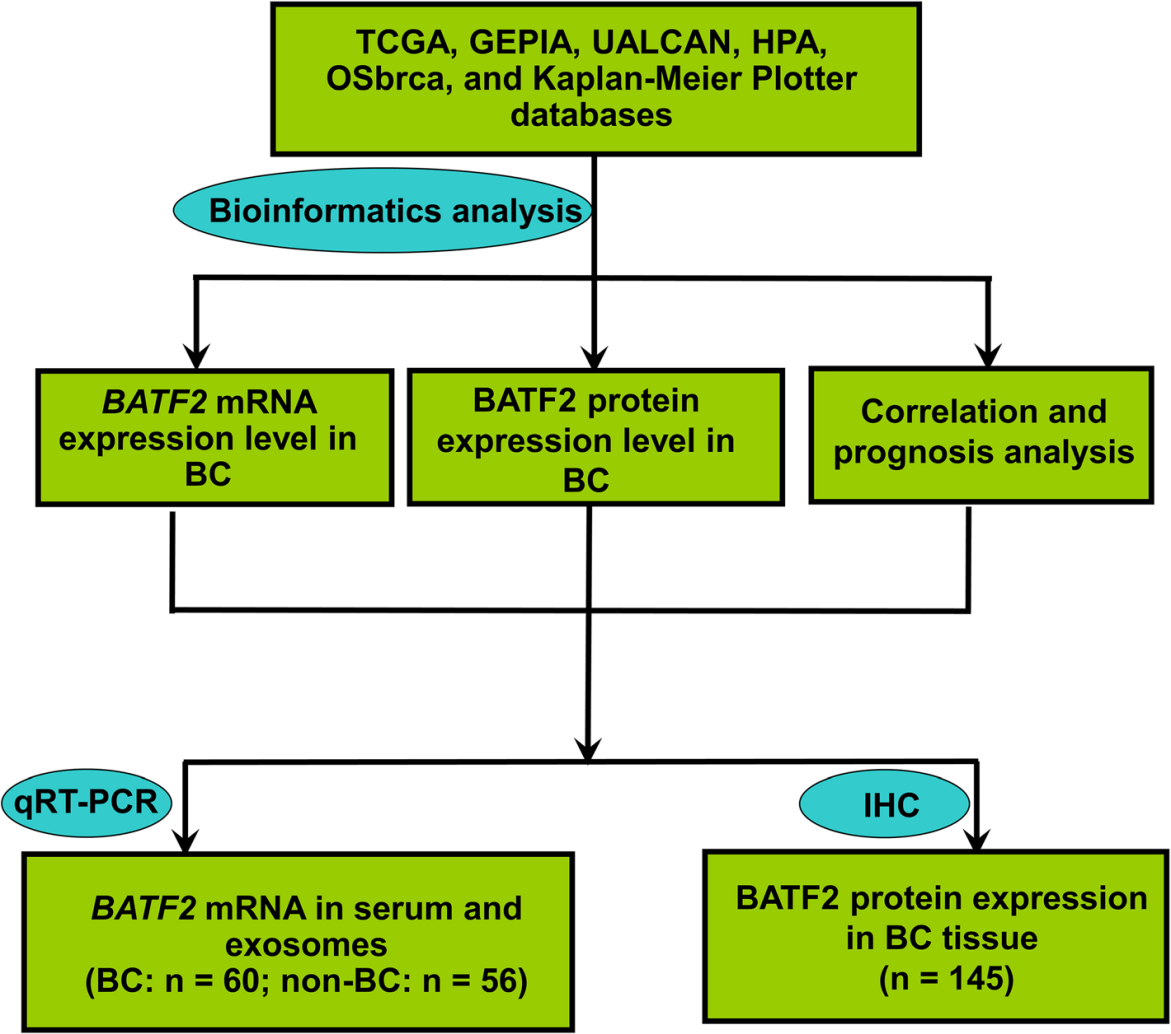
Flow chart of the study design

**Fig. 2:**
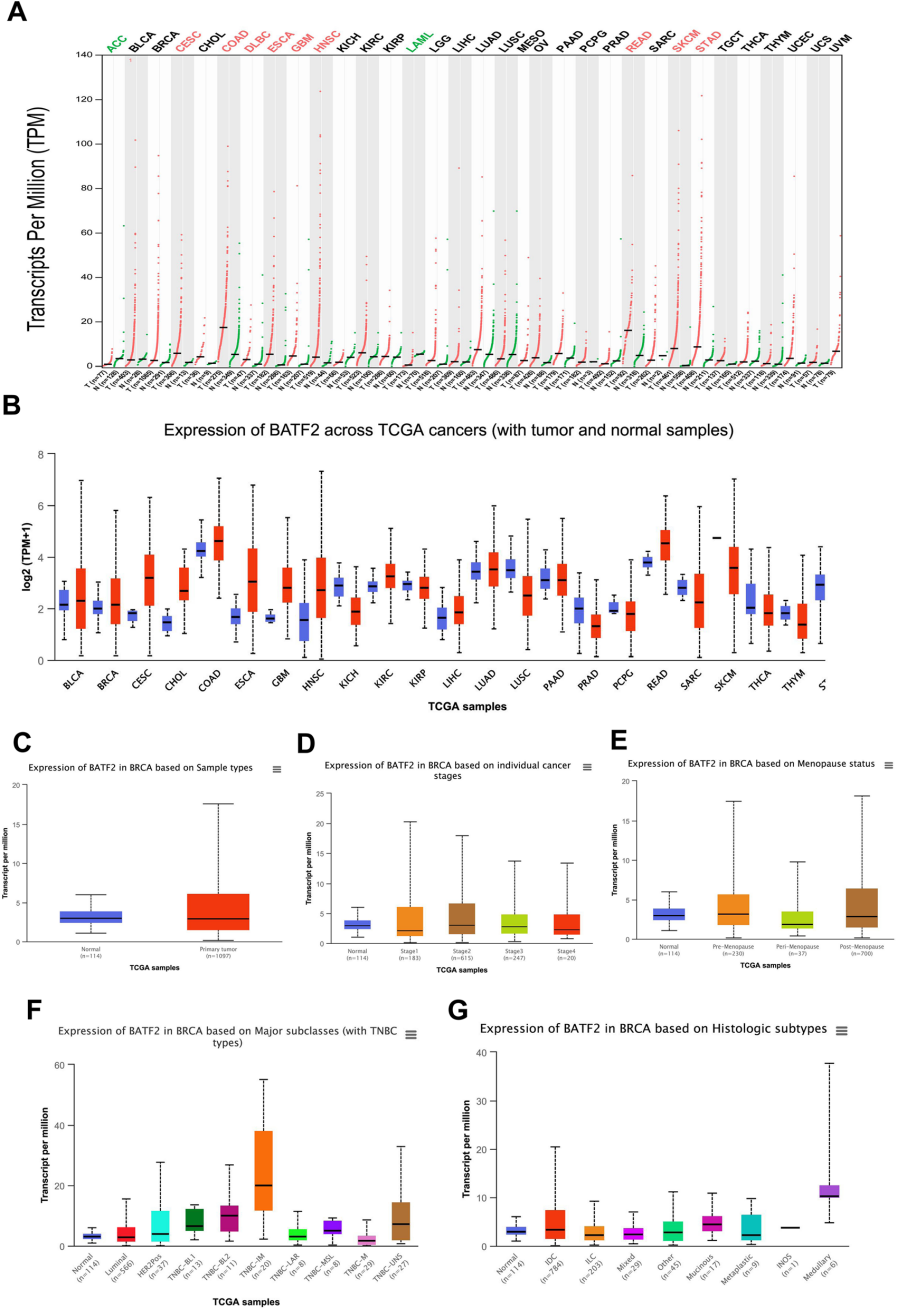
Expressions of *BATF2* mRNA in cancers analyzed based on GEPIA and UALCAN databases. Pan-cancer view of *BATF2* mRNA in (**A**) GEPIA and (**B**) UALCAN databases. Expressions of *BATF2* mRNA in BRCA cases based on (**C**) sample types, (**D**) cancer stages, (**E**) menopause status, (**F**) subclasses, and (**G**) histologic subtypes

**Fig. 3:**
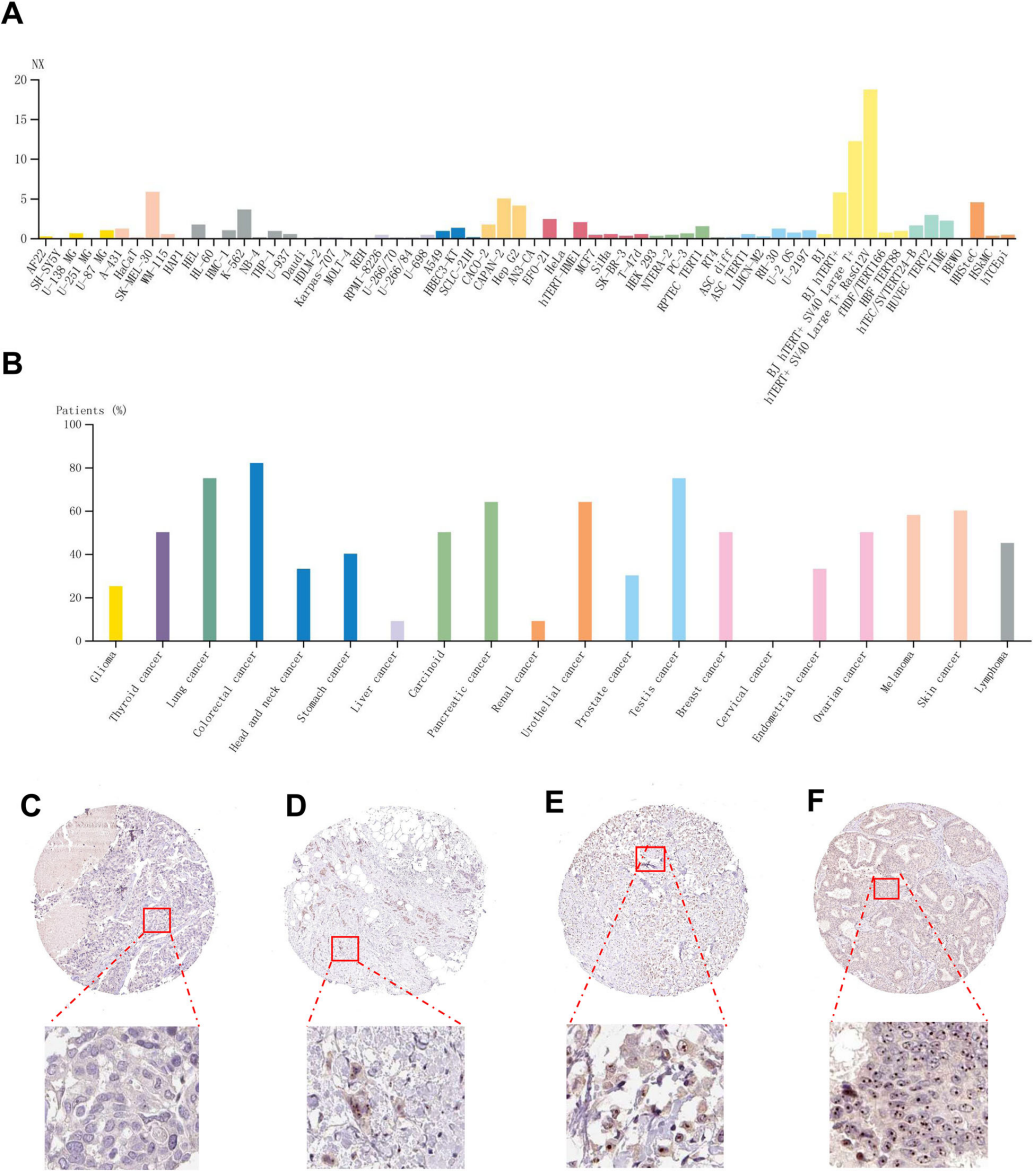
Expressions of BATF2 mRNA and protein in cancers analyzed based on HPA database. (**A**) Pan-cancer view of *BATF2* mRNA in cell lines. (**B**) Pan-cancer view of BATF2 protein expression in cancer patients. (**C**-**F**) Expression of BATF2 in BC tissues by IHC (× 400)

All procedures of this study design were depicted in Fig. 1. The pan-cancer analysis showed that the expression level of *BATF2* mRNA in breast invasive carcinoma (BRCA) was slightly lower than that in normal controls, but without a significant difference (Fig. 2A and B). Comparisons of *BATF2* mRNA expression between distinct cancer stages, histologic subtypes, major subclasses, and menopause versus non-menopause status in BRCA were respectively assessed and summarized in Fig. 1C-G. Specifically, *BATF2* mRNA expression pronouncedly increased in patients with TNBC-IM (*n* = 20) (Fig. 2F), or medullary BC (Fig. 2G). Besides, BATF2 protein expression (using HPA data) also detectable in cancer cell lines and cancer tissues (Fig. 3A and B), which was tested positive in more than 58.33% (7/12) of the BRCA patients in the HPA data. Among the positive samples of the HPA data, a moderate level (Staining: medium; Intensity: moderate; Quantity: > 75%) of BATF2 protein expression appeared in 5 cases, and a low level (Staining: low; Intensity: weak; Quantity: 75–25%) of BATF2 expression was shown in 2 cases (Fig. 3C-F shows the expression status of BATF2 in 4 BRCA cases).

### BATF2 interaction network and correlation analysis

**Fig. 4:**
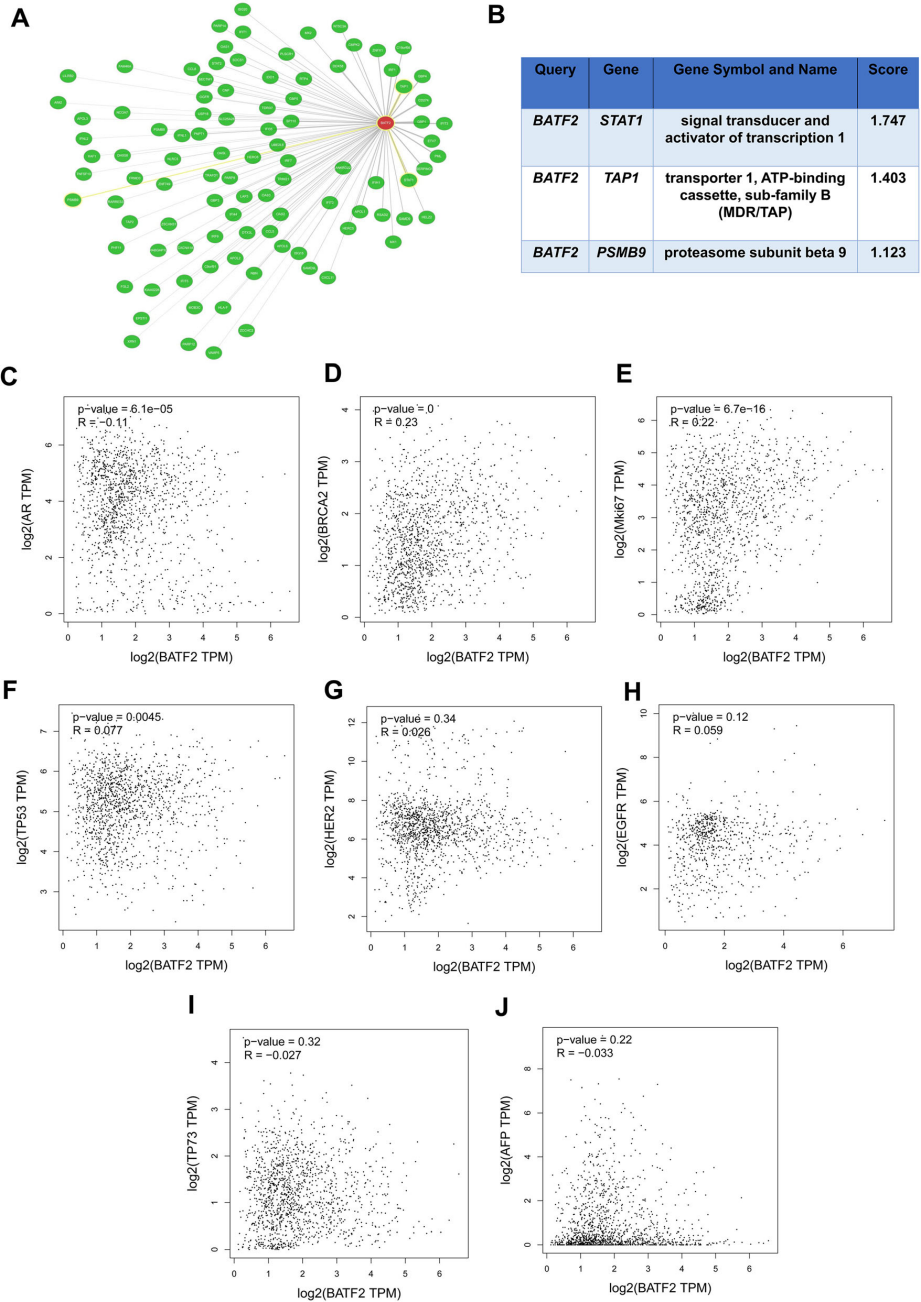
Co-expression networks and correlation analysis of *BATF2* utilized Coexpedia tool. (**A**) Co-expression networks of *BATF2* in Pan-cancer. (**B**) Co-expression genes with *BATF2* in BC. Correlation analysis of *BATF2* mRNA with (**C**) *AR*, (**D**)*BRCA2*, (**E**)*Mki67*, (**F**)*TP53*, (**G**)*HER2*, (**H**) *EGFR*, (**I**)*TP73*, and (**J**) *AFP* based on GEPIA database

The gene co-expression network of *BATF2* mRNA in Pan-cancer was plotted using Coexpedia, as shown in Fig. 4A. Finally, 98 genes were predicted to interact with *BATF2* mRNA, and the co-expression of *BATF2* with *TAP1*, *STAT1*, and *PSMB9* was the most critical in the occurrence and development of BC (Fig. 4B). Based on GEPIA database, *BATF2* mRNA expression was negatively correlated with *AR* expression (Fig. 4C) and positively associated with *BRCA2* (Fig. 4D), *Mki67* (Fig. 4E), and *TP53* (Fig. 4F) expressions (all with *P* < 0.01). There were non-significant associations between *BATF2* expression and *HER2*, *EGFR*, *TP73*, or *AFP* (Fig. 4G-J) (all with *P* > 0.05).

### *BATF2* mRNA expression has little influence on BC prognosis

**Fig. 5:**
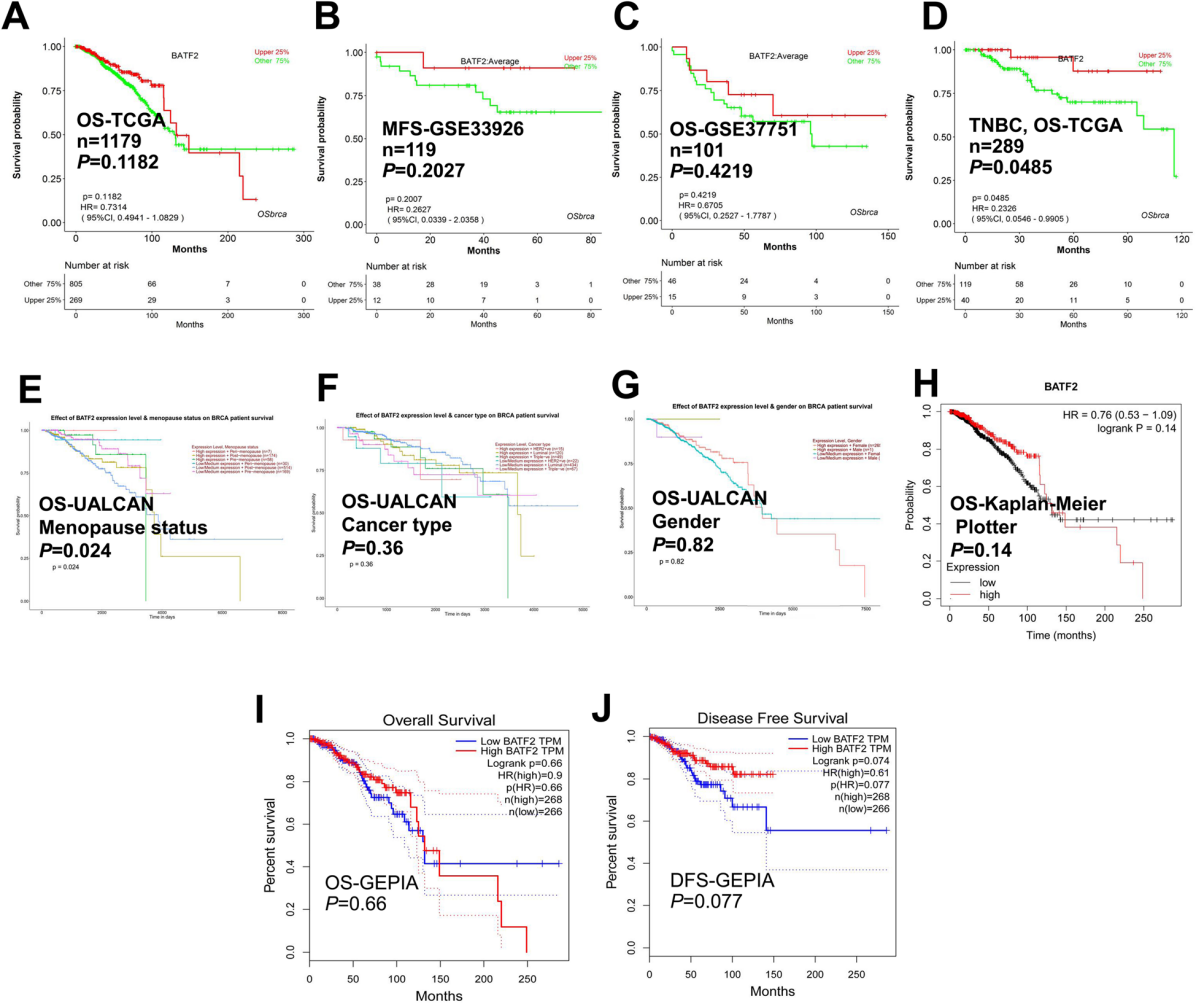
Survival analysis of *BATF2* mRNA in BC based on (**A**, **B**, **C**, **D**) OSbrca, (**E**, **F**, **G**) UALCAN, (**H**) Kaplan-Meier Plotter, and (**I**, **J**) GEPIA databases. OS: overall survival; MFS: metastasis-free survival; DFS: disease-free survival

Based on different datasets in OSbrca, *BATF2* mRNA expression displayed no significant influence on BC prognosis (Fig. 5A-C). However, the subgroup analysis showed that the overall survival (OS) of TNBC patients with a high *BATF2* mRNA level was significantly longer than that of patients with a low *BATF2* mRNA level (*P* = 0.0485; Fig. 5D). Based on UALCAN datasets, high *BATF2* expression level and menopause status were indicators of a better prognosis of BRCA patients (Fig. 5E) (cancer type and gender were not significant indicators, see Fig. 5F and G). However, there were non-significant correlations between *BATF2* mRNA expression and the survival of BRCA patients from Kaplan-Meier Plotter and GEPIA databases (Fig. 5H-J).

### IHC validation for the prognostic value of BATF2 protein expression in BC

**Fig. 6:**
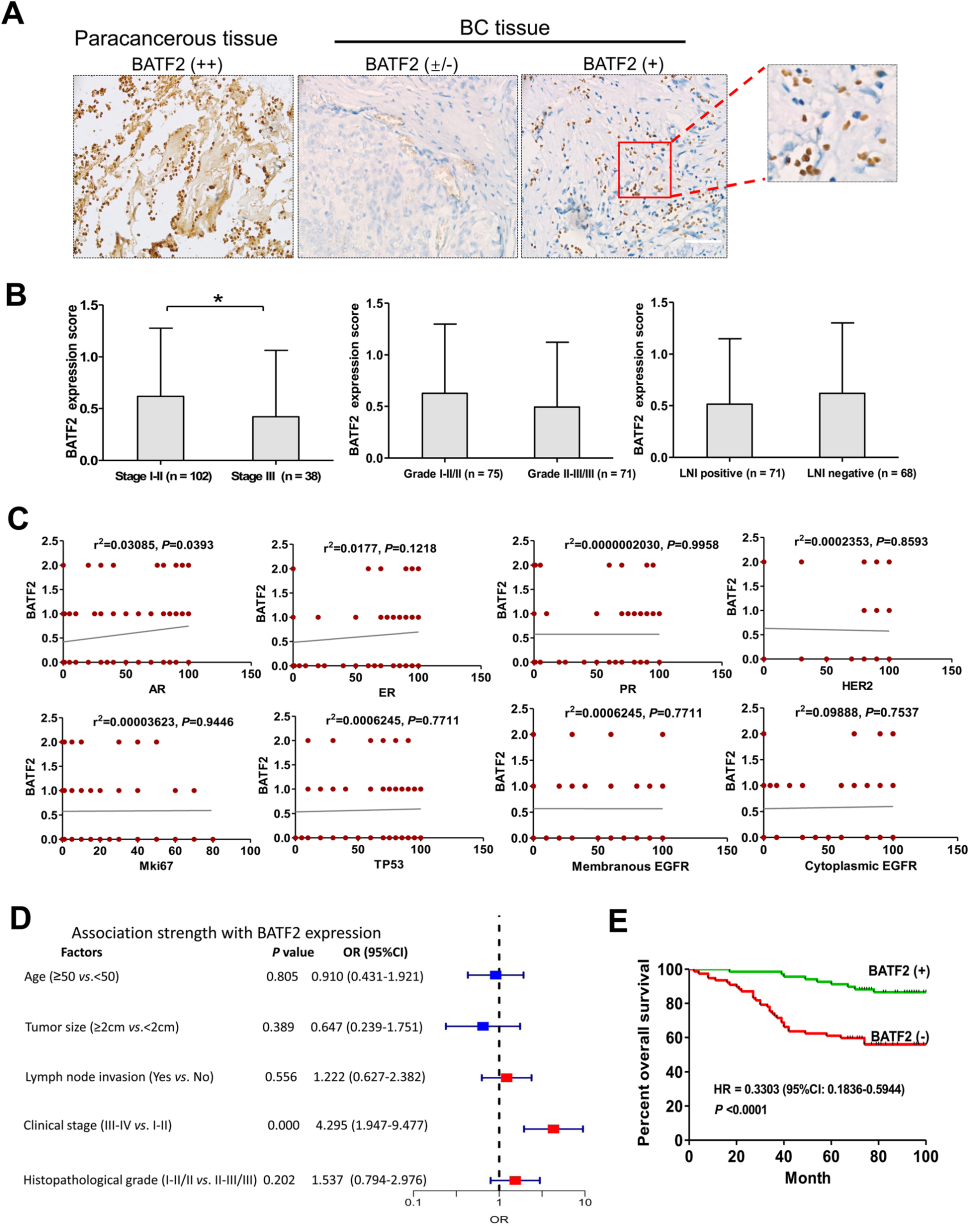
External validations of BATF2 protein expression in BC tissues by IHC. (**A**) Expression of BATF2 in BC and paracancerous tissues (× 200). (**B**) BATF2 level (rating score) in BC patients grouped by the status of clinical stage, pathology grade and lymph node invasion (LNI). (**C**) correlation analysis of BATF2 protein expression with AR, ER, PR, HER2, Mki67, TP53 and EGFR. (**D**) Analysis of the association strength between BATF2 expression and clinicopathological parameters in BC. (**E**) The plotted survival curve of BATF2 protein in predicting the OS of BC cases (*n* = 145). *P < 0.05

We further detected BATF2 protein expression in cancerous and adjacent noncancerous tissues in BC using high-throughput tissue microarray data and validated the expression using IHC analysis. The results showed that BATF2 was mainly expressed in the nucleus of BC cancer cells (Fig. 6A). Of 145 patients, 68 were positive for BATF2 expression, with a positive rate of 46.90%. Of the 68 positive individuals, only 19.17% (13/68) showed a moderate expression level (+), and the remaining were with a low level (weak staining, ±). The positive rate of BATF2 expression in adjacent noncancerous tissues reached 62.22% (56/90). Besides, BATF2 expression (rating scores) were higher in patients with stage I-II than those with stage III (Fig. 6B). BATF2 protein expression was correlated with clinical stage (*P* < 0.0001; Table 1) and AR expression (*P* = 0.0393; Fig. 6C), rather than estrogen receptor (ER), progesterone receptor (PR), HER2, Mki67, TP53 and EGFR (all with *P* > 0.05; Fig. 6C). Correlation analysis showed that BATF2 protein level was related to clinical stage (OR = 4.295, 95%CI: 1.947–9.477, *P* = 0.000; Fig. 6D). The survival analysis showed that BATF2 protein expression was positively associated with BC prognosis: the OS of patients positive for BATF2 expression was significantly prolonged compared to those negative for BATF2 expression (HR = 0.3303, 95%CI: 0.1836–0.5944; *P* < 0.0001; Fig. 6E). These results suggest that BATF2 can be used as a prognostic indicator of BC.

**Table 1:** Correlations between BATF2 expressions and clinical characteristics in BC patients

Clinicopathological features	Total cases	BATF2 expression (+)	BATF2 expression (−)	χ2 value	*P* value
Age (years)	145			0.061	0.805
≥ 50	108	50	58		
< 50	37	18	19		
Tumor size	141			0.743	0.389
≥ 2 cm	123	55	68		
< 2 cm	18	10	8		
Lymph node invasion	139			0.348	0.556
Yes	69	34	35		
No	70	31	39		
Clinical stage	140			13.964	< 0.0001
Stage I + II	94	54	40		
Stage III	46	11	35		
Histopathological grade	143			1.631	0.202
I-II/II	74	39	35		
II-III/III	69	29	40		

### Serum and exosomal *BATF2* mRNA expressions in BC patients

**Fig. 7:**
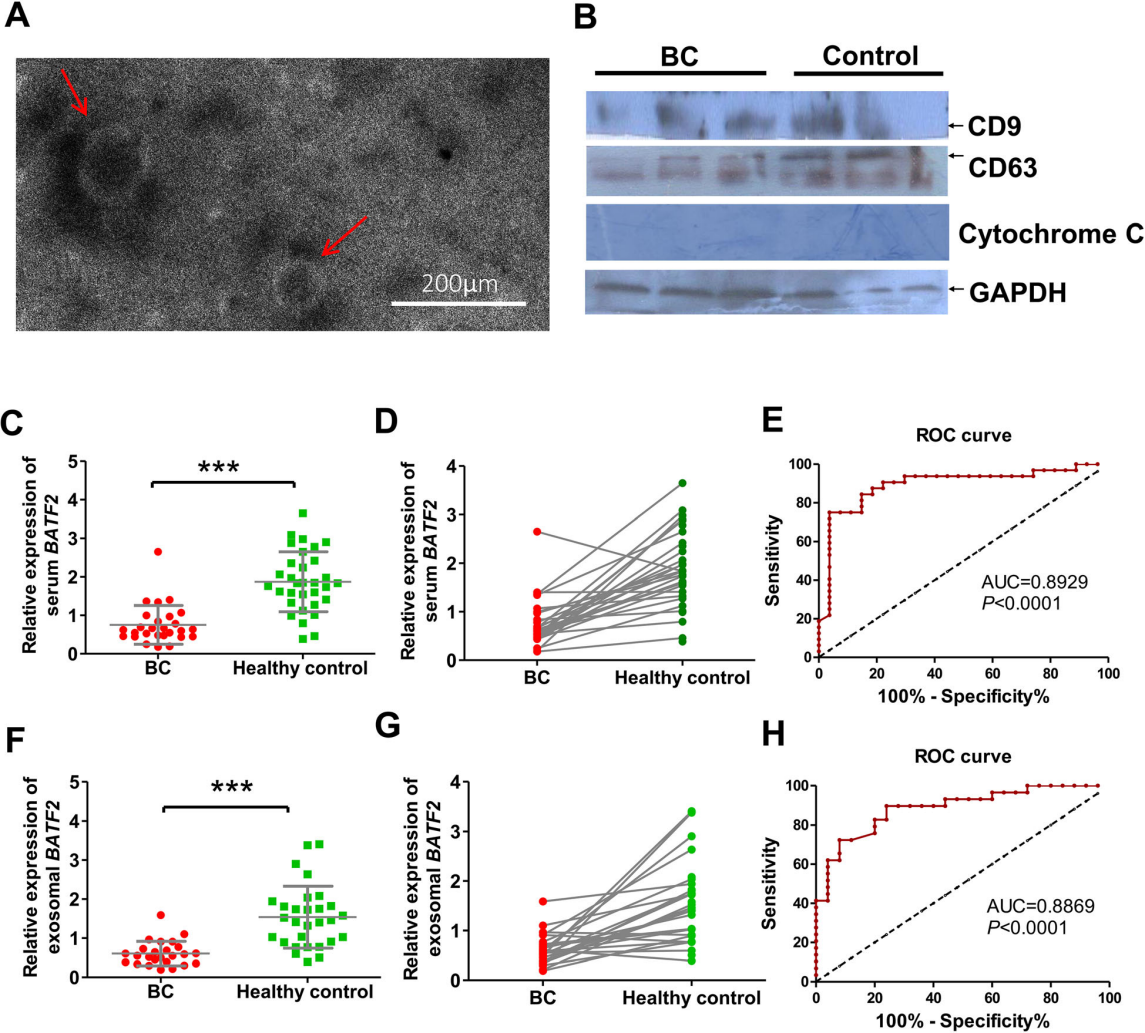
The tumor-specific pattern of *BATF2* expression in serum and exosome in BC cases. (**A**) Morphology of serum-sourced exosomes under TEM. The red arrows indicate isolated vesicles. (**B**) Detection of exosomal marker proteins as CD9 and CD63 in isolated vesicles by immunoblotting. Cytochrome C (mitochondrial marker) was used as a negative control to exclude the possible mixture of cellular contamination. (**C**, **D**) The expression level of *BATF2* mRNA in BC serum and the (**E**) plotted ROC curve used to identify BC. (**F**, **G**) Decreased expression of serum-derived exosomal *BATF2* in BC patients, and (**H**) the ROC curve of exosomal *BATF2* in confirming BC. ***P < 0.0001

The morphology and size of serum-derived exosome vesicles were assessed using the TEM examination. TEM images revealed serum exosomes were tiny, 30–150 nm vesicles with a membrane structure (Fig. 7A). They were further identified by determining the expressions of exosomal marker proteins CD9 and CD63 using western blot analysis (Fig. 7B). CD9 and CD63 expressions were detectable in the eluent, whereas Cytochrome C expression was negative (a negative control to exclude the possible mixture of cellular contamination), indicating that serum exosomes were successfully extracted. The qRT-PCR assay showed that the positive rates of serum and exosomal *BATF2* mRNA expressions were 45.00% (27/60) and 41.67% (25/60) in BC patients, respectively, versus the positive rates of 57.14% (32/56) and 51.80% (29/56) in healthy controls. Serum *BATF2* mRNA expressions were down-regulated in BC cases (Fig. 7C and D). For serum *BATF2* mRNA expression, ROC curves yielded a sensitivity of 84.38%, a specificity of 85.19% (Youden index = 0.6956), and the AUC of 0.8929 for BC diagnosis from healthy individuals (Fig. 7E). Similarly, exosomal *BATF2* mRNA expressions were also decreased in BC cases (Fig. 7F and G). Exosomal *BATF2* mRNA expression exhibited an AUC of 0.8869 for BC diagnosis (Fig. 7H), with a sensitivity of 82.76% and a specificity of 80.00% (Youden index = 0.6275). These results suggest that serum and exosomal *BATF2* mRNA expressions are promising indicators for BC diagnosis.

## Discussion

The occurrence of breast cancer (BC) is a female-prevalent malignancy with massive involvement of intricate proto-oncogene networks and tumor suppressor gene inactivation [3–5]. Current evidence supports *BATF2* as a tumor suppressor gene in various malignancies. Su ZZ et al. first reported that BATF2 overexpression in malignant glioma, melanoma, and prostatic cancer cell lines strongly inhibited the growth and apoptosis of cancer cells, without harm to the survival of noncancerous cells [6]. Ma H et al. found that the low BATF2 expression was positively correlated with the occurrence and development of liver cancer, and strikingly, all patients with down-regulated BATF2 expression had a poor prognosis [11]. Li et al. reported that BATF2 expression in prostate cancer was significantly associated with clinicopathological features such as serum PSA levels, clinical stage, and distant metastasis, which could be a critical player in the recurrence and progression of prostate cancer [26]. Consistently, other studies also suggest that a low BATF2 level is a risk factor for the poor prognosis in non-small cell lung cancer; BATF2 deletion promotes the EMT process, leading to LUAD cell invasion and metastasis [15]. *BATF2* mRNA expression was also significantly down-regulated in cancerous tissues of colorectal cancer: patients negative for BATF2 protein expression often exhibit a poor grade of tumor differentiation, deep invasion, a higher TNM-stage, and a short period of postoperative survival, with significant correlations [10]. The study of CML showed that CML patients often had lower serum *BATF2* mRNA expression levels than healthy individuals; the down-regulation of *BATF2* gene expression is related to *BCR-ABL* inhibition and participates in the occurrence and development of CML [8]. These mentioned studies imply that BATF2 can be used as a prognostic indicator of patients, a monitoring sensor for tumor therapy, and a potential target in gene therapy.

This work initially assessed BATF2 mRNA and protein expressions as diagnostic and prognostic biomarkers in BC using bioinformatics. Subsequently, these expressions and their clinical implications were fully confirmed in the serum, exosome, and cancer tissue samples of BC patients using qRT-PCR and IHC analyses. In the first step, we comprehensively analyzed *BATF2* mRNA and protein expressions using the expression data from GEPIA and UALCAN and HPA data from TCGA and GTEX. We found the expressions of *BATF2* mRNA and protein in BC tissues were at low-to-moderate levels. The average expression level of *BATF2* mRNA in healthy controls was slightly higher than that in BC cancer tissues, but there was no statistical difference. By contrast, BATF2 protein was mainly located in the nucleus of BC cancer cells based on HPA analysis, with a low-to-moderate level in protein expressions in 7 cases out of 12 cases. The correlation analysis revealed that *BATF2* mRNA was co-expressed with *TAP1*, *STAT1*, and *PSMB9* in BC. Based on the GEPIA database, *BATF2* mRNA expression was negatively associated with the AR expression and positively correlated with *BRCA2*, *Mki67*, and *TP53* expressions, with non-significant relationships with *HER2*, *EGFR*, *AFP* and *TP73* expressions. Some studies have confirmed that BATF2 expression is negatively correlated with CCN1 expression and regulates the biological behaviors of cancer cells via regulating CCN1 expression in vivo [9].

We further evaluated the relationship between the differentially expressed *BATF2* mRNA levels and BC prognosis in UALCAN [19], OSbrca [21], Kaplan-Meier Plotter [22], GEPIA [18], and other databases. Most databases yielded a non-significant correlation between *BATF2* mRNA expression and the prognosis, but the subgroup analyses uncovered the significantly prolonged OS of TNBC patients with high *BATF2* mRNA expressions versus the low expression group. *BATF2* mRNA expression levels and menopause status were also associated with the survival of BRCA patients. However, information on BATF2 expression and other biomarkers for BC prognosis is currently needed. In external validations, we determined the expression and prognostic value of BATF2 in BC patients using tissue microarray and IHC analysis. The results showed that BATF2 was mainly located in the nucleus of cancer cells of BC, which was consistent with the results of HPA analysis, yet with a lower positive rate of BATF2 protein expression. And all validation samples positive for BATF2 protein showed its expressions at low-to-moderate levels (according to the IHC staining score). Correlations analysis showed that BATF2 protein expression was positively correlated with AR expression, which is in line with the correlation analysis results based on GEPIA. The survival analysis based on tissue microarray data showed that patients with high BATF2 expressions had a longer OS. Therefore, a high BATF2 expression in BC can be a protective factor for the prognosis of patients. However, the survival analysis of BATF2 expressions in HPA database showed that BATF2 expression (high =254 versus low = 821) yielded a *P* value of 0.053 in predicting the OS of the breast invasive carcinoma patients. Further investigations are still needed to verify the prognostic significance of BATF2 in BC.

Exosomes are extracellular nanovesicles (30-150 nm) fabricated via a series of regulatory processes, as simplified by “endocytosis - fusion - exocytosis” [26]. Recent studies have shown that exosomes act as carriers containing miRNA, mRNA, DNA fragments, proteins, and other bioactive substances, involving in various physiological and pathological processes [27, 28]. It has been proven that exosomes are enriched in the peripheral blood, urine, saliva, ascites, amniotic fluid, and other body fluids; and that tumor-derived or tumor-related exosomes even participate in the regulation of tumor occurrence and development [29]. The quantification of tumor exosomes can assist in early diagnosis, curative effect evaluation, and the prognosis of tumor patients [30]. Our previous studies reported that the clinical implications of serum and exosomal *LDHC* gene (a CTA molecule) expressions in BC and HCC, serving as an assistant for diagnosis, efficacy evaluation, and recurrence monitoring [23, 24]. In BC patients, the positive rates of *BATF2* mRNA expressions in the serum and exosomes were 45.00 and 41.67%, respectively, versus 57.14 and 51.80% in healthy controls. Both serum and exosomal *BATF2* showed the AUCs for BC diagnosis of higher than 0.85, which exhibited promising diagnostic values. Similarly, Roe JK et al. reported the *BATF2* transcript level as a single sensitive biomarker in differentiating active pulmonary and extracellular TB from healthy individuals [30]. Our study, for the first time, confirmed the expression and diagnostic value of serum and exosomal *BATF2* in BC, providing preliminary evidence for further research on the clinical application of BATF2 in BC patients.

Besides, we discovered that the prognosis assessment based on BATF2 tissue microarray did not yield the results of *BATF2* mRNA as a molecular index for BC prognosis prediction. Several explanations can be considered. Firstly, the survival analysis was conducted based on BATF2 protein expressions using IHC scores. Secondly, *BATF2* mRNA expression levels can be inconsistent with its protein levels due to protein posttranslation modifications, and different analyses for protein and mRNA expressions may also contribute to inconsistent results. It is reported that the linear relationship between mRNA and protein expression levels is only about 0.4 to 0.5. For instance, a study by Antonis Koussounadis et al. reported that there was merely a weak correlation between all their measured mRNA and protein expression levels (r = 0.08, *n* = 579, *P* = 0.07) [31]. Thus, different measurement analyses, statistical methods, and expression levels cohesively result in inconsistent results.

## Conclusions

This study demonstrates the down-regulation of BATF2 mRNA and protein expressions in BC and their diagnostic and prognostic implications in part of BC subtypes based on bioinformatics. Further clinical validations using serum and exosome samples have confirmed the results, which suggest that BATF2 is expected to be a new molecular marker for BC diagnosis and prognosis assessment. However, the sample size in this study is insufficient, which may inevitably bias the conclusion to some extent. Therefore, more validations incorporating large sample data are needed to further confirm the findings in this study.

## Supplementary Information

**Additional file 1.**

## Data Availability

The datasets used and/or analyzed during the current study are available from the corresponding author upon reasonable request.

## Abbreviations

AR: Androgen receptor
BATF2: Basic leucine zipper ATF-like transcription factor 2
BC: Breast cancer
BRCA2: BRCA2 DNA repair associated
CCN1: Cellular communication network factor 1
CML: Chronic myeloid leukemia
ESCC: Esophageal squamous cell carcinoma
EMT: Epithelial-mesenchymal transition
HPA: Human Protein Atlas
HCC: Hepatocellular carcinoma
IHC: Immunohistochemistry
IFN: Type I interferon
LUAD: Lung adenocarcinoma
Mki67: Marker of proliferation Ki-67
NSCLC: Non-small-cell lung cancer
OS: Overall survival
qRT-PCR: Real-time quantitative reverse transcription polymerase chain reaction
TP53: Tumor protein p53
TNBC: Triple-negative breast cancer
