# Supplementary figures and images for: Expression and clinical implications of basic leucine zipper ATF-like transcription factor 2 in breast cancer
Source: BMC Cancer. 2021 Sep 26;21:1062. doi: 10.1186/s12885-021-08785-6 (PMC8474811; doi:10.1186/s12885-021-08785-6)

CD9


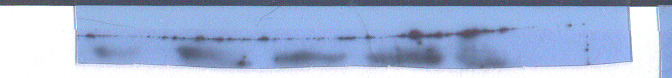


CD63


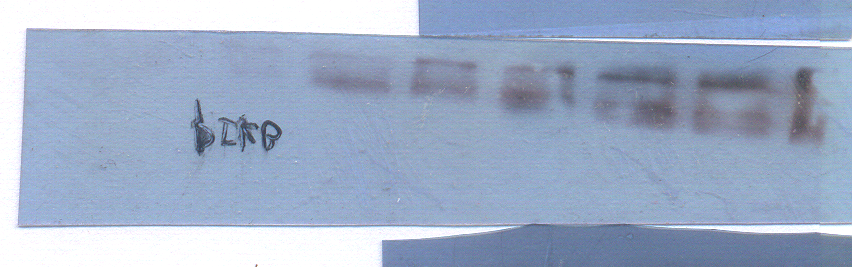


GAPDH


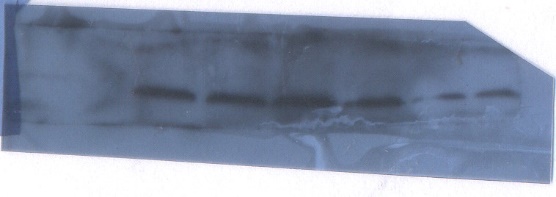


Cytochrome C


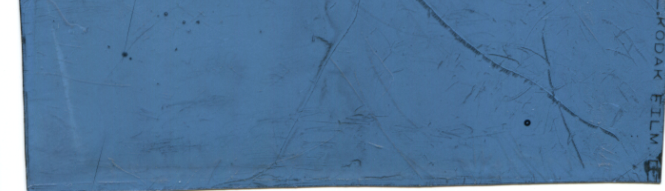

Supplement: Supplementary file 1 — Additional file 1. [file 12885_2021_8785_MOESM1_ESM.docx]
